# Miracles persist: a reply to Sus

**DOI:** 10.1007/s13194-021-00440-y

**Published:** 2022-02-25

**Authors:** Niels Linnemann, James Read

**Affiliations:** 1 University of Bremen, Bremen, Germany; 2 University of Oxford, England, UK

**Keywords:** Relativity, Noether’s theorem, Symmetries, Dynamical approach

## Abstract

In a recent article in this journal, Sus purports to account for what have been identified as the ‘two miracles’ of general relativity—that (1) the local symmetries of all dynamical equations for matter fields coincide, and (2) the symmetries of the dynamical equations governing matter fields coincide locally with the symmetries of the metric field—by application of the familiar result that every symmetry of the action is also a symmetry of the resulting equations of motion. In this reply, we argue that, while otherwise exemplary in its clarity, Sus’ paper fails in this regard, for it rests upon a illegitimate application of the aforementioned result. Thus, we conclude, *pace* Sus, that these two miracles persist in general relativity.

## Introduction

In a recent article in this journal Sus (2021) argues that what Read et al. (2018) dub the ‘two miracles’ of general relativity—that (1) the local symmetries of all dynamical equations for matter fields coincide, and (2) the symmetries of the dynamical equations governing matter fields coincide locally with the symmetries of the metric field—are after all derivable from innocuous assumptions on the matter sector of that theory. In Sus’ words,

the Einstein field equation constrains the equations for the non-gravitational fields by imposing that, insofar as such fields are *sources* for the gravitational fields, such equations must be locally Poincaré invariant, and the motion of *force-free bodies* is approximately geodesic. This provides content to the attempts at explaining the constraints of the non-gravitational laws by the metric, carried out in the [geometrical approach], in a way that it does not presuppose any mysterious a priori connection and that is relevant to accounting for the miracles. (Sus, 2021, p. 16)

The purpose of this response article is to demonstrate that Sus’ argument does not go through as intended; as we will argue, he makes illegitimate assumptions regarding the action of the matter sector from whose (local) invariance under the Poincaré transformations the (local) Poincaré invariance of the matter field equations is taken to follow. In particular, Sus appeals to the familiar result that every variational symmetry (i.e., symmetry of the action) is also a dynamical symmetry (i.e., symmetry of the equation of motion) (See, for instance, Brading (2001) and Doughty (2018, Section 9.3)). However, as we will see, Sus applies this result to the on-shell action (i.e., assuming the equations of motion to hold), whereas in fact the result holds only for the off-shell action (i.e., without assuming the equations of motion to hold). This turns out to be fatal for Sus’ purported derivation of the miracles.

In the remainder of this paper, we elaborate on this in detail. In Section 2, we expose the lacuna in Sus’ reasoning regarding the symmetry theorem. In Section 3, we give an explicit example demonstrating that Sus’ inferences fail.

There is one further comment which we should make before we begin. One might legitimately raise the concern that all work on the ‘two miracles’ of general relativity is stymied by the lack of precision in these discussions—in particular, regarding the notion of ‘local symmetries’ which these discussions deploy (See, for example, Weatherall (2021, Section 3.1) for a pointed critique in this regard). Although in our view appeal to recent work such as that of Fletcher (2020) can go some way to resolving these issues, for the purposes of this note we do not engage with these discussions: rather, our focus lies solely on Sus’ argument, as presented in Sus (2021), and we accept the shared premises of Read et al*.* and Sus—in particular, regarding the meaningfulness of the centrally-employed notion of ‘local symmetry’ in the sense of form-invariance of mathematical structures defined at each point of the manifold under specific coordinate transformations, such as those of the Poincaré group.^1^

## A problematic inference

Recall that the metric field equations and matter field equations of general relativity can be obtained by extremising the action $S = S_G + S_M$, where $S_G = \int R \sqrt{-g} d^4 x$ and $S_M = \int \mathcal {L}_M \left( g_{ab}, \phi ^i \right) \sqrt{-g} d^4 x$. $S_G$ is, of course, the Einstein-Hilbert action. Recall also that the variation of $S_M$ reads

1 $$\begin{aligned} \delta S_M= & {} \int \frac{\delta ( \mathcal {L}_M \sqrt{-g})}{\delta g_{ab}} \delta g_{ab} d^4 x + \int \frac{\delta \mathcal {L}_M}{\delta \phi _i} \delta \phi _i \sqrt{-g} d^4 x \nonumber \\= & {} \int \left( \frac{\delta \mathcal {L}_M}{\delta g_{ab}} + \frac{1}{2} g^{ab} \mathcal {L}_M \right) \delta g_{ab} \sqrt{-g} d^4 x + \int \frac{\delta \mathcal {L}_M}{\delta \phi _i} \delta \phi _i \sqrt{-g} d^4 x . \end{aligned}$$

Sus’ strategy is to derive the Poincaré invariance of the equations of motion for the matter fields $\phi _i$ via the following steps:

1. Assume that the equations of motion for (all) $\phi _i$ hold. Thereby, the second term in (1) can be dropped and we obtain
  2 $$\begin{aligned} \delta S_M^{\text {on-shell for }\phi _i} = \int T^{ab} \delta g_{ab} \sqrt{-g} d^4 x, \end{aligned}$$
  where the definition of the Hilbert stress-energy tensor
  3 $$\begin{aligned} T^{ab} := \frac{\delta \mathcal {L}_M}{\delta g_{ab}} + \frac{1}{2} g^{ab} \mathcal {L}_M\end{aligned}$$
  has been used. (There are three salient points to be made here. First, in the following, one should distinguish $S_M^{\text {on-shell for }\phi _i}$ and $S_M$. Second, Sus neglects the second term in the Hilbert stress-energy tensor (3); we further discuss this matter in Section 3 in the context of a consideration of the symmetries of (2). And third, locally, the Hilbert stress-energy tensor does not always coincide with a locally-derived Noether stress-energy tensor—see our discussion in §3.)
2. Show then that $\delta S_M^{\text {on-shell for }\phi _i}$ is (locally) invariant (up to boundary terms) under Poincaré transformations.
3. Infer from the premise that $\delta S_M^{\text {on-shell for }\phi _i}$ is (locally) invariant (up to boundary terms) under Poincaré transformations to the conclusion that the equations of motion for (all) $\phi _i$ are (locally) invariant under Poincaré transformations.

Although it is not the focus on our arguments in this paper, let us say something on Sus’ methodology in the second of these steps. It is first argued that matter fields which source the Einstein equation $G_{ab} = 8 \pi T_{ab}$ must have stress-energy tensors $T_{ab}$ which satisfy $\nabla ^{a} T_{ab} = 0$. This is taken to mean that there are ten locally conserved currents $T^{ab} \omega _b$, associated respectively with ten approximate local Killing vector fields $\omega ^b$ relative to $g_{ab}$—so that we have

4 $$\begin{aligned} \partial _a \left( T^{ab} \omega _b \right) = 0, \end{aligned}$$

for *any* normal coordinate system (note that, given that $\nabla$ is induced by a Lorentzian metric, there is at least a whole class of normal coordinate systems associated to one another via local Poincaré transformations).^2^

Sus alludes to using the converse of Noether’s first theorem at this stage to make statements about the symmetry group of the action:

... one can derive the transformation properties of an action integral from the existence of divergences of some quantities by applying the converse of Noether’s first theorem. This is how it would work out in this case. (Sus, 2021, p. 19)

*If* these ten currents are associated to the matter sector of the theory—i.e. the theory encoded by the action $S_M$—then one can indeed use the converse of Noether’s first theorem to assert the (local) invariance of $S_M$ under ten corresponding continuous (local) symmetries. Note that this would still not mean that these ten locally conserved currents are necessarily associated with the local Poincaré symmetry group; after all, the conventional wisdom that a conserved current (e.g. energy-momentum) is always connected to the same kind of symmetry transformation has been demonstrated by Smith (2009) to be false.

But it is in particular the antecedent of the above conditional claim which can be problematic. These ten currents seem to be associated to the matter sector if the metric $g_{ab}$ relative to which the Killing vectors in these currents—and thus the currents themselves—are defined is the unambiguous metric structure ‘seen’ by the matter sector. However, as we will see in Section 3, $g^{ae} \nabla _e T_{ab} = 0$ can obtain without the matter Lagrangian from which $T_{ab}$ is derived featuring the metric $g_{ab}$ in any straightforward sense, and moreover without the equations of motion exhibiting local Poincaré symmetries. In such a case, there is then no physical motivation to ascribe any physical meaning to (matter) currents obtained from Killing vectors that are associated to $g_{ab}$.

All of the above being said, one can argue by other means (and, indeed, Sus accomplishes this in Sus (2021, Section 5)—clearly, without using the converse of Noether’s first theorem) that $S_M^{\text {on-shell for }\phi _i}$ is locally Poincaré invariant. As we will continue to argue below, $S_M^{\text {on-shell for }\phi _i}$ does not contain the decisive information about the matter sector which would allow one to derive the (local) Poincaré invariance of the matter equations of motion; this constitutes a fatal blow to Sus’ argument.

In the third step above, Sus makes appeal to the statement that all symmetry transformations that leave the action invariant (up to boundary terms) also leave invariant all the equations of motion that follow via Hamilton’s principle from that action (Sus, 2021, fn. 44). However, showing via this statement that the equations of motion for the fields $\phi _i$ are (locally) Poincaré invariant requires showing the (local) invariance of *all* terms in the action $S_M$ involving $\phi _i$ under the Poincaré symmetry transformation—not just that of $S_M^{\text {on-shell for }\phi _i}$, i.e. not just of that part of $S_M$ which remains when the equations of motion for the $\phi _i$ are applied to the action (see e.g. Brading, 2001). In other words, one could say that Sus’ above reasoning involves an illegitimate step between (2) and (3), namely:

2.5. Infer from the premise that $\delta S_M^{\text {on-shell for }\phi _i}$ is (locally) invariant (up to boundary terms) under Poincaré transformations to the conclusion that $\delta S_M$ is (locally) invariant (up to boundary terms) under Poincaré transformations.

Heuristically speaking, the problem then lies in disregarding the fact that the information about the equations of motion (including their symmetry structure) is first and foremost to be found in their corresponding action term (i.e., the action terms from which the equations of motion of interest actually follow from a variational principle—which would be $S_M$ in the given case, rather than $S_M^{\text {on-shell for }\phi _i}$). To make the point as tangible as possible, we provide in Section 3 a clear counterexample to the soundness of step (3). Before doing so, however, we demonstrate in the remainder of this section some difficulties regarding other perhaps *prima facie* innocuous assumptions and claims made in Sus’ paper.

An important passage in Sus’ article comes in footnote 39, where he recognises that “...one could restrict further the symmetries of matter laws through the introduction of fixed fields that break local Poincaré invariance”, but questions whether the resulting theory “would be GR anymore” (Sus, 2021, fn. 39). If theories featuring fixed fields are precluded by fiat, then since Sus is also dealing with general relativity—and, hence, theories with a Lorentzian metric field appearing in the associated action principles—one might think that, *by stipulation*, Sus is dealing with theories in which the off-shell action has local Poincaré symmetries, and in turn (after varying the action) that $\delta S_M^{\text {on-shell for }\phi _i}$ is invariant locally under Poincaré transformations.^3^

We have seen that step (2.5) is a problematic inference in general. Given the above, however, we are now in a position to see that Sus has (at least on this reading) in fact sought to secure the consequent (*viz*., the local Poincaré invariance of $\delta S_M$) by other means—i.e., by *stipulating* that theories featuring fixed fields in their action are precluded. But this, in turn, allows us to conclude two things. First: Sus’ reasoning via the symmetry theorem (as presented in Section 2), is a red herring: what he seeks to demonstrate is, in fact, *assumed*—albeit implicitly—in footnote 39. Insofar as this (the thought might go) precludes by fiat miracle-violating scenarios, one could argue that Sus has, in fact, *not* derived these miracles within the framework of general relativity; rather, he too presupposes them.

In fact, however, this reasoning itself is problematic—for the preclusion of fixed fields in a theory with a Lorentzian metric field $g_{ab}$ appearing in its action does *not* necessarily yield local Poincaré invariance—think of a case in which terms in action also couple to an unfixed but timelike vector field $\lambda ^a$; such a theory will be discussed explicitly in Section 3.^4^ Thus, the correct thing to say here, ultimately, is this: Sus’ reasoning regarding the symmetry theorem fails; moreover, the restrictions made in footnote 39 are still insufficient to secure that which Sus is after.

Finally, even if it were the case that the assumptions made in that footnote were sufficient to secure the miracles, there still seems to be room to argue (potentially with Sus) that general relativity, correctly understood, indeed assumes (if only implicitly) the contents of footnote 39: one could maintain that the preclusion of fixed fields is a kinematical constraint of general relativity, delimiting the range of what is possible according to that theory. Proponents of the ‘dynamical view’ promulgated by Brown and Pooley (see Brown, 2005; Brown & Pooley, 2001, 2006) are unlikely to be convinced—for their concern is this: why is it the case, in the *actual, physical world*, that material fields are such that they all obey dynamical equations of a certain kind? From the point of view of these theorists, abstract modal talk of the kinematical possibilities according to some theory does nothing to resolve this *physical* question.

In sum: in our view, Sus’ work does not afford the possibility of ‘relativity without miracles’, for (a) the specific reasoning deployed in his paper—proceeding via the implicit assumption of (2.5)—is flawed on technical grounds, (b) the assumptions presented in footnote 39 of Sus’ paper might be seen as begging the question by (the thought might go) assuming the content of the miracles, and (c) even then, these assumptions are, in fact, insufficient to guarantee the miracles. There remains one further point to be made in this article. In the interests of clarity, it would be valuable to present a theory which demonstrates the illegitimacy of the inference in (2.5), as presented in Section 2. Such a theory we now present.

## A problem case for Sus’ argument

We consider a ‘Newtonian’ variant of the Jacobson-Mattingly theory (which in turn was introduced in Jacobson & Mattingly (2001), and later exposed to the philosophical literature in Read et al. (2018, Section 6)). First recall that the equation of motion for the gravitational potential $\varphi$ of Newtonian gravity is the Newton-Poisson equation,

5 $$\begin{aligned} h^{ab}\nabla _a \nabla _b \varphi = 4 \pi G_N \rho , \end{aligned}$$

where $h^{ab}$ is a degenerate metric field of signature $\left( 0,1,1,1\right)$ (Malament, 2012, ch. 4); for simplicity in the ensuing, we will set Newton’s constant $G_N = 1$, and will also assume a constant and normalised matter density content ($\rho = 1$)—although the latter is a significant restriction, this will not compromise the point which we seek to make in this section. Now, letting $\lambda ^{a}$ be some unfixed normalised timelike vector field, recall that we can define $h^{ab}$ in terms of the (inverse) metric field $g^{ab}$, as

6 $$\begin{aligned} h^{ab} = g^{ab} + \lambda ^a \lambda ^b . \end{aligned}$$

Treating $h^{ab}$ in (5) as shorthand for (6), and moreover interpreting $\varphi$ as a matter sector scalar field, we can write down the matter sector Lagrangian as

7 $$\begin{aligned} \mathcal {L}_{M} = \frac{1}{2} h^{ab} \nabla _a \varphi \nabla _b \varphi + 4 \pi \varphi , \end{aligned}$$

where $\nabla$ is the Levi-Civita derivative operator associated with $g_{ab}$. Since $\lambda ^a$ is unfixed, this action is still diffeomorphism invariant (see footnote 4); accordingly, the response equality $\nabla ^a T_{ab} = 0$ (‘conservation of energy-momentum’) obtains.^5^ Indeed, this covariant conservation of the stress-energy tensor can be shown explicitly—to do so, note first that the equations of motion for $\lambda ^a$ and $\varphi$ are, respectively,

8 $$\begin{aligned} \lambda ^a \nabla _a \varphi \nabla _b \varphi= & {} 0, \end{aligned}$$

9 $$\begin{aligned} \nabla _a \left( h^{ab} \nabla _b \varphi \right)= & {} 4 \pi ; \end{aligned}$$

in particular, (8) implies $\lambda ^a \nabla _a \varphi = 0$, so $\nabla _a \varphi$ is orthogonal to $\lambda ^a$. Furthermore, the Hilbert stress-energy tensor can be computed to be

10 $$\begin{aligned} T_{ab} = \frac{1}{2} \nabla _a \varphi \nabla _b \varphi - \frac{1}{4} g_{ab} h^{cd} \nabla _c \varphi \nabla _d \varphi - \frac{1}{2} g_{ab} (4 \pi ) \varphi . \end{aligned}$$

Using (8)-(10), one can thereby compute:

11 $$\begin{aligned} g^{ea} \nabla _e T_{ab}= & {} \frac{1}{2} g^{ea} \left( \nabla _e \nabla _a \varphi \right) \nabla _b \varphi + \frac{1}{2} g^{ea} \nabla _a \varphi \nabla _e \nabla _b \varphi - \frac{1}{2} g_{ab} g^{ea} g^{cd} \left( \nabla _e \nabla _c \varphi \right) \nabla _d \varphi \nonumber \\&- \frac{1}{4} g_{ab} g^{ea} \nabla _e \left( \lambda ^c \lambda ^d \right) \nabla _c \varphi \nabla _d \varphi - \frac{1}{2} g_{ab} g^{ea} \lambda ^c \lambda ^d \left( \nabla _e \nabla _c \varphi \right) \nabla _d \varphi \nonumber \\&- \frac{1}{2} g_{ab} g^{ea} \left( 4 \pi \right) \nabla _e \varphi \nonumber \\= & {} \frac{1}{2} \left( g^{ea} \nabla _e \nabla _a \varphi - 4 \pi \right) \nabla _b \varphi - \frac{1}{4} \nabla _b \left( \lambda ^c \lambda ^d \right) \nabla _c \varphi \nabla _d \varphi \nonumber \\&- \frac{1}{2} \lambda ^c \lambda ^d (\nabla _b \nabla _c \varphi ) \nabla _d \varphi \nonumber \\= & {} 0 . \end{aligned}$$

where both equations of motion (8) and (9) were used in the final step.

In this theory, the presence of the timelike vector field $\lambda ^a$ means that locally this Lagrangian is invariant not under the Poincaré group, but rather under the Leibniz group (see Pooley (2013, Section 3.1) for an explicit presentation of this group). Indeed, the matter action $S_M = \int \mathcal {L}_M \sqrt{-g} d^4x$ is also invariant under Leibniz transformations, as locally the volume element takes the form of the unimodular condition $\sqrt{-g} = 1$; one can compute explicitly that this condition is preserved under Leibniz transformations. This (and only this) Leibniz invariance obtains also for the equation of motion (5) derived from the matter action via a variational principle.

At the same time, one can compute

12 $$\begin{aligned} \delta S_M^{\text {on-shell for } \varphi , \lambda ^a} = \frac{1}{2} \int \left( \nabla ^a \varphi \nabla ^b \varphi + g^{ab} \mathcal {L}_M \right) \delta g_{ab} \sqrt{-g} d^4x. \end{aligned}$$

In particular, note that (12) is invariant under local Poincaré transformations. After all, for local Poincaré transformations, one has $\delta g_{ab} = \nabla _{(a} \omega _{b)}$ where $\omega ^a$ is one of the ten approximate local Killing vector fields associated with $g_{ab}$. Then, one has

13 $$\begin{aligned} \delta S_M^{\text {on-shell for } \varphi , \lambda ^a}= & {} \frac{1}{2} \int \left( \nabla ^a \varphi \nabla ^b \varphi + g^{ab} \mathcal {L}_M \right) ^{\text {on-shell for } \varphi , \lambda ^a} \nabla _{(a} \omega _{b)} \sqrt{-g} d^4x \nonumber \\= & {} 0, \end{aligned}$$

where the term vanishes because $\omega ^a$ is a (local) Killing field. But this means that, according to the selfsame methodology presented in Sus (2021, pp. 19-20), the equations of motion for $\varphi$ would have to count as (locally) Poincaré invariant—something which is evidently not the case in this example: for recall (5).

A final word on the theory currently under consideration. This constitutes an example of a theory for which a locally-defined gauge-invariant Noetherian stress-energy tensor and the localised Hilbertian stress-energy tensor do not even agree up to a constant factor (cf. related examples in the context of Minkowski spacetime considered in Baker et al. (2021)): Locally, the Hilbert stress-energy tensor for the theory under consideration is

14 $$\begin{aligned} T^{\text {Hilbert, local}}_{\mu \nu } = \frac{1}{2} \partial _\mu \varphi \partial _\nu \varphi - \frac{1}{4} \eta _{\mu \nu } \partial _i \varphi \partial ^i \varphi - \frac{1}{2} \eta _{\mu \nu } (4\pi ) \varphi , \end{aligned}$$

where $i, j, k, \ldots$ range only over spatial indices. By contrast, using that $\mathcal {L}_M^{\text {local}} = \frac{1}{2} \partial _i \varphi \partial ^i \varphi +4\pi \varphi$, we obtain the following expression for the Noether stress-energy tensor:

15 $$\begin{aligned} \frac{1}{2} T^{\text {Noether, local}}_{\mu \nu }= & {} \frac{1}{2} \frac{\partial \mathcal {L}_M^{\text {local}}}{\partial (\partial ^\mu \varphi )} \partial _\nu \varphi - \frac{1}{2} \eta _{\mu \nu } \mathcal {L}_M^{\text {local}} \nonumber \\= & {} - \frac{1}{2} \delta _{0\mu } \delta _{0\nu } \partial _0 \varphi \partial _0 \varphi + \frac{1}{2} \partial _\mu \varphi \partial _\nu \varphi - \frac{1}{4} \eta _{\mu \nu } \partial _i \varphi \partial ^i \varphi - \frac{1}{2} \eta _{\mu \nu } (4\pi ) \varphi . \end{aligned}$$

Notably though, both $T^{\text {Hilbert, local}}_{\mu \nu }$ and $T^{\text {Noether, local}}_{\mu \nu }$ are conserved just in case one of them is conserved, as they agree on-shell up to a constant factor. More concretely, requiring the local version of the equation of motion for $\lambda ^a$ to hold—i.e., requiring that $\partial _0 \varphi = 0$—is sufficient to show equality up to a constant factor.^6^

## Closing remarks

Sus’ paper constitutes perhaps the best attempt yet to provide an account of the miracles of general relativity from within the framework of that theory. If Sus’ project were successful, then we agree that it would offer “a richer perspective” on the miracles (Sus, 2021, fn. 61). However, its success is cast into doubt by the fact that Sus makes an incorrect inference regarding the variational symmetry theorem (Section 2). As Sus himself concedes, this implies that their miraculous status continues to obtain (“... seeing the coincidence of all the symmetries of laws as a mere coincidence [is an option which] must be taken when there is no dynamically determined interaction that can be used to derive the constraints” (Sus, 2021, p. 27)). At least to us, it is not clear how the above-identified issues with Sus’ argument can be overcome; in the absence of such a resolution, it remains the case that the best accounts of the miracles of general relativity (and ones which would be acceptable to proponents of the dynamical approach) lie in successor theories—such as spin-2 gravity (Salimkhani, 2020), or theories of quantum gravity (Read, 2019).

## Footnotes

1 See the appendix of Read et al. (2018) for details.

2 Here (to fill in some details absent in Sus’ discussion at Sus (2021, p. 18)), one can regard $\partial _a$ as a ‘coordinate derivative operator’—see Malament (2012, p. 64). If one prefers, one could make the same point by writing (4) in coordinates.

3 Read et al. (2018), Sus (2021), and ourselves all make use of the notion of a fixed field presented by Pooley in (Pooley 2017, p. 115)—such an object is a field fixed identically in all kinematical possibilities of a theory. In addition, all three parties can be taken to accept the understanding of diffeomorphism invariance presented in Pooley (2017, p. 117), for which the salient transformations are those which transform dynamical fields, but not fixed fields. For further details on all these assumed background notions, see Pooley (2017) and Read (2016, 2020b).

4 Notably, such a condition does not have to violate the diffeomorphism invariance of the theory in question, for the space of kinematical possibilities $\langle M, g_{ab}, \lambda ^a , \Phi \rangle$ of some theory $\mathcal {T}$, where $\Phi$ is a placeholder for material fields, will partition into equivalence classes under diffeomorphisms in which the vector field $\lambda ^a$ is timelike, spacelike, or null. By contrast, *fixing* $\lambda ^a$ privileges a representative of (some) of the above-mentioned equivalence classes, and thereby breaks the diffeomorphism invariance of the theory.

5 See Brown and Brading (2001, Section 5) for an elaboration on the relationship between diffeomorphism invariance and the covariant conservation of the stress-energy tensor. Of course, the response equation can also be shown straightforwardly to obtain using the matter field equations of motion, as discussed in the main text to follow. Finally, note that whether this response equation truly represents conservation of energy-momentum is a difficult question: see e.g. Hoefer (2000); Lam (2011); Pitts (2010) and Read (2020a) for recent philosophical discussion.

6 It would be interesting to explore further the connection between (a) theories in which the Noetherian stress-energy tensor comes apart from the Hilbertian stress energy tensor and (b) theories in which Sus’ argument fails.
